# *In Vitro* Cytotoxicity of GuttaFlow Bioseal, GuttaFlow 2, AH-Plus and MTA Fillapex

**DOI:** 10.22037/iej.v12i3.15415

**Published:** 2017

**Authors:** Gokhan Saygili, Suna Saygili, Ibrahim Tuglu, Ismail Davut Capar

**Affiliations:** a *Department Of Endodontics, Izmir Katip Celebi University, Faculty of Dentistry, Turkey; *; b *Depatment of Histology and Embryology, Faculty of Medicine, Celal Bayar University, Manisa, Turkey; *; c *Private Practice, Istanbul, Turkey*

**Keywords:** AH-Plus, Cytotoxicity, GuttaFlow Bioseal, GuttaFlow 2, MTA Fillapex, MTT Assay, TUNEL Assay

## Abstract

**Introduction:**

The aim of the present *in vitro* study was to evaluate the cytotoxicity of different sealers including GuttaFlow Bioseal, GuttaFlow 2, AH-Plus and MTA Fillapex on L929 murine fibroblasts.

**Methods and Materials:**

Samples of GuttaFlow Bioseal, GuttaFlow 2, AH-Plus and MTA Fillapex were fabricated in Teflon disks of 5 mm diameter and 3 mm thickness. L929 fibroblasts were exposed to the extracts of these materials for 3, 24, 72 and 168 h at 37^°^C with 5% CO_2_. Cell viability was evaluated by the 3-(4, 5-dimethylthiazol-2-yl)-2, 5-diphenyltetrazolium bromide (MTT) assay. Apoptosis was evaluated by the terminal deoxynucleotidyl transferase-mediated dUTP nick-end labeling (TUNEL) assay. The data were analysed by ANOVA.

**Results:**

GuttaFlow Bioseal was nontoxic at all experimental time points (*P*>0.05), whereas MTA Fillapex and AH-Plus were toxic (*P*<0.001). At 7 days, there were more viable cells in the GuttaFlow 2 group than in the control group, and MTA Fillapex was more cytotoxic than AH-Plus. There were more apoptotic cells in the MTA Fillapex and AH-Plus groups than in the other groups at 3 h (*P*<0.001).

**Conclusion:**

GuttaFlow sealers are less cytotoxic than MTA Fillapex and AH-Plus. At all experimental time points, there was no significant difference in the cell viability between the GuttaFlow Bioseal group and the control group.

## Introduction

Root canal sealer covers dentin tubules and prevents infection of the root canals. If it overflows onto the periapical area, it should not be toxic to the hard or soft tissues [1]. The content of root canal sealers is important because some of the sealers cause a reaction in the tissue and increase tissue inflammation [2, 3]. 

AH-Plus (Dentsply, DeTrey, Konstanz, Germany) contains an epoxy resin and was found to be cytotoxic due to minimal release of formaldehyde [4, 5]. Although MTA Fillapex (Angelus, Londrina, PR, Brazil) contains MTA (Mineral Trioxide Aggregate), there are conflicting results regarding its biocompatibility, due to the presence of toxic components, such as salicylate resin, diluting resin and silica [6, 7]. GuttaFlow 2 (Roeko-Coltène/Whaledent, Langenau, Germany) is a silicone-based root canal sealer. The particle size of its powder form is less than 30 µm, and it contains gutta-percha powder, poly dimethyl siloxane, platinum catalyst, zirconium dioxide and micro-silver. Previous studies have shown that the biocompatibility of Gutta Flow 2 is higher than that of AH-Plus [8, 9].

**Figure 1 .**
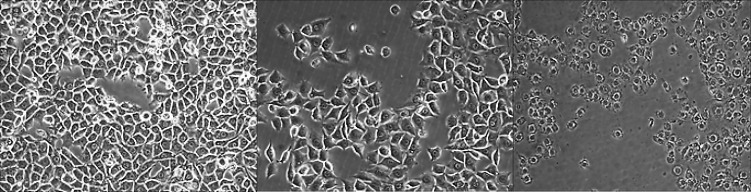
*Representative phase contrast images of: *A)* control; *B)* moderate where half of the cells were death; and *C)* severe toxic effect where most of the cells were dead on the L929 Fibroblast (10× magnification). There was cell death morphology with apoptosis which cells showed nuclear condensation and blebbing*

Recently, GuttaFlow Bioseal (Coltène/Whaledent AG, Altstatten, Switzerland) has been introduced. It contains some bioactive substances, such as calcium and silicate, which the manufacturer says stimulate tissue regeneration and healing. The working and curing time of GuttaFlow Bioseal is shorter than that of GuttaFlow 2 and it also combines free-flow gutta-percha with an appropriate sealer at room temperature according to manufacturer’s instructions [10].

To observe the long-term biocompatibility of root canal sealers, retrospective and primarily controlled prospective clinical studies in humans should be performed. However, *in vitro* cellular studies can be useful in providing information about the biological properties of new materials [11, 12]. Therefore, the cytotoxicity of this sealer in comparison to AH-Plus and MTA Fillapex was assessed in the present study.

## Materials and Methods

***Cell culture***

A mouse fibroblast cell line (L929, Sap Institute-Republic of Turkey Ministry of Food Agriculture and Livestock Eskisehir) was routinely cultivated in Dulbecco's Modified Eagle’s Medium (DMEM, F0445, Biochrom, Berlin, Germany) supplemented with 10% fetal bovine serum (FBS ,S0113, Biochrom, Berlin, Germany), 100 UI/mL penicillin and 100 UI/mL streptomycin (A2213, Biochrom, Berlin, Germany), 200mM L-glutamin (K0282, Biochrom, Berlin, Germany) at 37^°^C and 5% CO_2_. Cells were seeded at 30000 cells/well in 24-well plates and incubated for 24 h at 37^°^C.

***Sample preparation and extraction procedures***

All materials [GuttaFlow Bioseal (Roeko-Coltène/Whaledent, Langenau, Germany), GuttaFlow 2 (Roeko-Coltène/Whaledent, Langenau, Germany), MTA Fillapex (Angelus, Londrina, PR, Brazil) and AH-Plus (Dentsply, Tulsa Dental, Tulsa, OK, USA)] were mixed according to the manufacturers’ instructions. The compositions of these materials are shown in Table 1. Each sealer was mixed under aseptic conditions. Then sealers were placed in pre-sterilised cylindrical Teflon disks with 5 mm diameter and 3 mm thickness (Applied Plastics Technology, Inc, Bristol, RI, USA). The materials were kept to set at 37^°^C in a humidified atmosphere with 5% CO_2_ for 24 h before extraction. Extracts of the materials were prepared in 24-well dishes by immersing them in DMEM cell culture media supplemented with 10% FBS, penicillin and streptomycin and incubated in the dark 37^°^C at 3, 24, 72 and 168 h. The 200 µL extracts were diluted 1:1 with culture media for the testing. The cells were exposed to extracts for 24 h. Control group including only culture medium were treated similarly.

***Cytotoxicity assay***

Extracts were sterilized by a 0.22 µm filter (Merck Millipore, Billerica, MA, USA). Pure DMEM medium was used as negative control and cells without extracts were used as positive control. Fibroblast morphology and the effects of extracts from root canal sealers were observed under an inverted phase contrast microscope (Olympus, model IX50, Japan) with magnification ratio of 10:1. The 30000 cells in the 24-well culture dish were exposed to 400 µL culture media containing extracts for different time points such as 3, 24, 72 and 168 h and then, the medium was removed without washing. Cell survival was determined using the 3-(4, 5-dimethylthiazol-2-yl)-2,5-diphenyltetrazolium bromide (MTT, M6494, Invitrogen, USA) assay. MTT solution (0.5 mg/mL) was added to each well, and cells were incubated for an additional 4 h. The resulting formazan crystals were dissolved when removing the culture medium and adding dimethyl sulfoxide solvent (Sigma-Aldrich Biotechnology, St. Louis, MO, USA) to each well. The plates were shaken at room temperature for 10 min to dissolve the crystals and were then taken to the reader. The enzyme inhibition quantification was measured using a spectrophotometer (ELx800UV, Biotek, USA) at 570 nm. Four replicate cell cultures were exposed to each of the extract serial dilutions in three independent experiments. The absorbance readings were normalised to untreated control cultures. All experiments were repeated three times.

***TUNEL assay***

Apoptosis was determined by enzymatic labelling of DNA strand breaks using the terminal deoxynucleotidyl transferase-mediated deoxyuridine triphosphate nick-end labelling (TUNEL) assay. TUNEL labelling was conducted using the *in situ* Cell Death Detection Kit conjugated with horseradish peroxidase (TUNEL, S7101, Millipore, USA) and performed according to the manufacturer's instructions. Briefly, 30000 L929 cells grown on sterile Lab-Tec chamber slides were incubated with extracts of GuttaFlow Bioseal, GuttaFlow 2, MTA Fillapex and AH-Plus at IC50 doses for different time points. After fixation with 4% paraformaldehyde for 30 min, slides were incubated with permeabilization solution (0.1% Triton X-100 in 0.1% sodium citrate) for 8 min at 4^°^C. After washing twice with PBS for 5 min, the labelling reaction was performed using the terminal deoxynucleotidyl transferase end-labelling cocktail for each sample (except the negative control, in which reagent without the enzyme was added) and incubated for 1 h at 37^°^C. For signal conversion, slides were incubated with 50 μL converter-horseradish peroxidase (prepared according to the manufacturer's instructions) for 30 min at 37^°^C, rinsed with PBS and then incubated with 50 μL 3,3′-diaminobenzidine (DAB) substrate solution (DAKO K3468, USA) for 10 min at 25^°^C. TUNEL-positive cells were examined and photographed using a Leica DM6000B microscope (BX43, Olympus, Japan) with a DC490 digital camera (SC50, Olympus, Germany). Apoptotic index were used to evaluate quantitative data. All experiments were repeated three times.

***Statistical analysis***

MTT and TUNEL results were evaluated using GraphPad software (GraphPad Instat v3.01, San Diego, CA, USA). Differences between median values were analysed by ANOVA test for comparisons among groups, with the level of significance set at 0.05.

## Results

***Fibroblast morphology***

**Figure 2.**
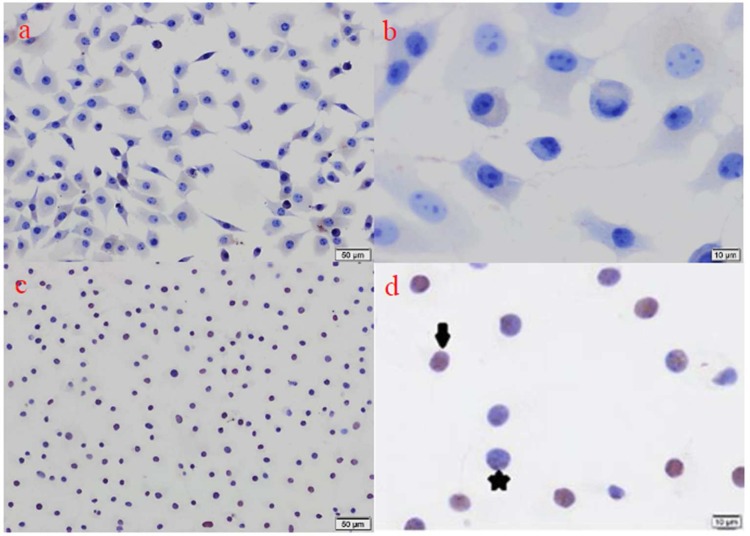
A and B)* Representative images of control cultures for TUNEL staining. None of the cell were positively labelled and all of them were alive; *C and D) Representative images of toxic cultures for TUNEL staining. approximately half of the cell were positively labeled. (↓: apoptotic cells with TUNEL, *: healty cells with TUNEL

The effects of extracts from root canal sealers were observed under an inverted phase contrast microscope, and changes in cell morphology were evaluated (Figure 1). In contrast to the control group, which had spindle-shaped cells that spread to all areas, the experimental groups, especially the MTA Fillapex group and the AH-Plus group at 3 h and 1 day, displayed rounded cells and decreased cell numbers. The apoptotic effects of extracts from endodontic sealers on L929 fibroblasts by TUNEL staining is shown in figure 2. There was clear and significant (*P*<0.001) apoptosis in the MTA Fillapex and AH-Plus groups starting from 3 h after application. 

**Table 1.** The composition of the test materials

**Material**	**Composition**	**Manufacturer**
**GuttaFlow Bioseal**	Gutta-percha, zinc oxide, barium sulfate, polydimethylsiloxane, bioactive glass ceramic, zirconia, platinum catalysis, color pigments, micro silver	(Coltene Whaledent, GmBH Co. KG, Langenau, Switzerland)
**GuttaFlow 2**	Gutta-percha powder, polydimethylsiloxane, silicone oil, paraffin oil, platinum catalyst, zirconium dioxide, micro silver (preservative), coloring	(Coltene Whaledent, GmBH Co. KG, Langenau, Switzerland)
**MTA Fillapex**	Salicylate resin, diluting resin, natural resin, bismuth oxide, nano particulated silica, MTA, pigments	Angelus (Londrina, PR, Brazil)
**AH-Plus**	Paste A: epoxy resins, calcium tungstate, zirconium oxide, silica, iron oxide pigments; Paste B: amines, calcium tungstate, zirconium oxide, silica, silicone oil	(Dentsply DeTrey, Konstanz, Germany)

**Table 2.** The mean (SD) of number of live cells from test groups by MTT; Similar letters indicate insignificant differences

**Time**	**Control**	**GuttaFlow 2**	**MTA Fillapex**	**AH-Plus**	**GuttaFlow Bioseal2**
**3 hours**	1082.55 (220.47)^ a^	1011.23 (220.56) ^a^	743.66 (212.22)^ a^	763 (201.44)^ b^	1074.74 (272.88)^ b^
**1 day**	1275.66 (172.22)^ a^	1212.12 (53.54) ^a^	852.98 (51.56)^ a^	744.22 (42.88)^ b^	1219 (180)
**3 days**	1221.56 (184.88)^ a^	1078.66 (54.77) ^a^	804.77 (52.54)^ a^	875.55 (63.77)^ b^	1146.88 (240)
**7 days**	1114.24 (172.36)^ a^	1058.33 (73.63)^ a^	877.66 (72.48)^ b^	916.12 (84.44)^ b^	1138.44 (200)

***Cytotoxicity assay***

The results of the MTT assay which represents live cell number by absorbents values are shown in Table 2. There was no significant difference in the number of viable cells between the GuttaFlow Bioseal group and the control group (*P*>0.05). GuttaFlow Bioseal was significantly less cytotoxic than AH-Plus and MTA Fillapex at all time points (*P*<0.001). It also showed the highest cell viability at 7 days, though there were no significant differences between GuttaFlow Bioseal and GuttaFlow 2 at the other time points (*P*>0.05). GuttaFlow 2 was less cytotoxic than AH-Plus and MTA Fillapex at all time points (*P*<0.001), except for AH-Plus at 7 days (*P*<0.05). There were no significant differences between AH-Plus and MTA Fillapex except for at 7 days, at which point MTA Fillapex was more cytotoxic than AH-Plus (*P*<0.05).

## Discussion

In the present study, the cytotoxicity of GuttaFlow Bioseal extracts was investigated on L929 murine fibroblast cells in comparison with that of other endodontic sealers for different time points. There was significant time dependent cytotoxic effect of endodontic sealers starting from 3 h. GuttaFlow Bioseal was more biocompatible than GuttaFlow 2, AH-Plus and MTA Fillapex.

To evaluate the biocompatibility of root canal materials, the cell culture technique has often been used. Testing materials for cytotoxicity in culture conditions is a useful method of evaluation prior to performing clinical studies. Cell culture studies may give clues as to the toxic component of a material. For example, previous studies [4, 5, 13] indicated that formaldehyde released from AH-Plus may be the reason for its cytotoxicity. It has been reported that formaldehyde release of AH-Plus is less than that of AH-26 (Dentsply, Tulsa Dental, Tulsa, OK, USA). Similarly, in the present study, AH-Plus was more cytotoxic than GuttaFlow sealers.

TUNEL is an *in situ* histological technique that reveals DNA fragments, which is indicative of apoptosis [14]. In previous studies, apoptosis was observed in pulp tissue [15], osteoblast cells [16] and dental pulp stem cells [17] using the TUNEL assay. In the present study, the TUNEL assay showed that AH-Plus and MTA Fillapex result in higher levels of apoptosis at 3 h than GuttaFlow 2 and GuttaFlow Bioseal.

GuttaFlow and GuttaFlow 2 consist of similar materials but in different proportions, and GuttaFlow 2 also contains silver particles. It has been reported that GuttaFlow 2 and GuttaFlow have similar biocompatibilities [9]. GuttaFlow Bioseal has two components that automatically mix bubble free, it is easy to use and it is based on silicone, such as GuttaFlow 2. The manufacturer claims that GuttaFlow Bioseal provides natural repair compounds, such as calcium and silicates that forms hydroxyapatite crystals when it comes into contact with fluids. To the best of our knowledge, only one study has been done on GuttaFlow Bioseal by Pereira *et al.* [18]. Akcay *et al.* [19] assessed dentinal tubule penetration by different root canal sealers, including GuttaFlow Bioseal, using laser scanning confocal microscopy. They showed that GuttaFlow Bioseal has similar dentinal tubule penetration to that of MTA Fillapex and AH-Plus. In our study, we showed that GuttaFlow Bioseal is significantly less toxic than AH-Plus and MTA Fillapex. It has previously been shown that AH-Plus and MTA Fillapex are cytotoxic in V79 fibroblasts and BALB/c 3T3 cells because they contain resin-based material [6, 20]. The biocompatibility of GuttaFlow Bioseal might be due to its bioactive content and its lack of resin [21].

Silicon is one of the main components of GuttaFlow 2 and GuttaFlow Bioseal. The main components of MTA are calcium oxide (CaO) and silicon besides silicates [22]. Nowadays, many of the biocompatible materials used for perforation repair, retrograde filling and regeneration treatment include silicates [5, 23-25]. GuttaFlow Bioseal differs from other GuttaFlow sealers as it also contains bioactive glass, which consists of silica, calcium oxide, sodium oxide and phosphorus oxide. Bioactive glass can be produced from soluble to non-resorbable and changed the proportions of them [26]. It has both osteo-integrative and osteo-conductive effects and bond mechanically to bone tissue through hydroxyapatite crystals [27]. It was suggested that calcium hydroxide is formed when CaO comes into contact with water [28]. Phosphorus ions play an important role in the formation of apatite crystals, and composed calcium phosphate is known to be a precursor of apatite [29, 30]. Future studies should assess whether the bioactive glass in GuttaFlow Bioseal has a positive effect on bone tissue. 

In case of MTA Fillapex contains paste formula (half of it) MTA particles, the cytotoxicity of MTA Fillapex was observed in stem cells and subcutaneous tissues [23, 31, 32]. It was reported that MTA Fillapex is extremely cytotoxic over a 2-week period [6]. Similarly, MTA Fillapex was highly cytotoxic in the present study. In this study, MTA Fillapex was more cytotoxic than AH-Plus at 7 days. Silva *et al.* [6] indicated that the cytotoxicity of MTA Fillapex is higher than that of AH-Plus in 3T3 fibroblast cells over a 4-week period. The cytotoxicity of MTA Fillapex may be due to its resin component such as diluting resin and natural resin. Our results showed the toxic effect and the best material for clinical use with the limitations of cell line culture; thus, more *in vivo* experiments are required. Further studies should be carried out to investigate the biological properties of GuttaFlow Bioseal in different stem cells and *in vivo*.

## Conclusion

Within the limits of the present study, it may be concluded that GuttaFlow 2 and GuttaFlow Bioseal are less cytotoxic in L929 mouse fibroblast cells than AH-Plus and MTA Fillapex. GuttaFlow Bioseal resulted in higher cell viability than GuttaFlow 2 at 7 days.
